# Treatment for illegal drug use disorders: the role of comorbid mood and anxiety disorders

**DOI:** 10.1186/1471-244X-14-89

**Published:** 2014-03-26

**Authors:** Maria Melchior, Elena Prokofyeva, Nadia Younès, Pamela J Surkan, Silvia S Martins

**Affiliations:** 1 Inserm, U1018, Centre for Research in Epidemiology & Population Health (CESP), Epidemiology of occupational and social determinants of health, F-94807 Villejuif, France; 2 University of Versailles Saint-Quentin, UMRS 1018, F-94807 Villejuif, France; 3 Université de Versailles Saint-Quentin EA 4047, Centre Hospitalier de Versailles, Le Chesnay, France; 4 Department of International Health, Johns Hopkins Bloomberg School of Public Health, Baltimore, MD, USA; 5 Department of Epidemiology, Columbia University Mailman School of Public Health, 10032 New York, NY, USA

**Keywords:** Illegal drug use disorder, Treatment, Unmet need for treatment, Mood disorders, Anxiety disorders, Epidemiology, General population sample

## Abstract

**Background:**

Our aim was to examine whether comorbid mood and anxiety disorders influence patterns of treatment or the perceived unmet need for treatment among those not receiving treatment for illegal drug use disorders.

**Methods:**

Data came from the National Epidemiologic Survey on Alcohol and Related Conditions (NESARC, 2001–2002 and 2004–2005, n = 34,653). Lifetime DSM-IV illegal drug use disorder (abuse and dependence), as well as comorbid mood (major depression, dysthymia, manic disorder, hypomanic disorder) and anxiety disorders (panic disorder, agoraphobia, social phobia, specific phobia, generalized anxiety) were ascertained by a standardized psychiatric interview. Treatment for illegal drug use disorders and perceived unmet need for treatment were assessed among individuals with illegal drug use disorder. Odds of treatment and odds of perceived unmet need for treatment were assessed using logistic regression, adjusting for socio-demographic characteristics, treatment for mood and anxiety disorders, and comorbid alcohol use disorder.

**Results:**

Out of 34,653 participants, 1114 (3.2%) had a diagnosis of lifetime illegal drug use disorder: 21.2% had a comorbid mood disorder only, 11.8% a comorbid anxiety disorder only, and 45.9% comorbid mood and anxiety disorders. Comorbid mood and anxiety disorders were not related to treatment for illegal drug use disorders but were associated with an elevated likelihood of unmet need for treatment: compared to participants with no comorbidities, multivariate ORs were 2.21 (95% CI: 1.23- 4.10) for mood disorder only, 2.38 (95% CI: 1.27-4.45) for anxiety disorder only, and 2.90 (95% CI: 1.71-4.94) for both mood and anxiety disorders.

**Conclusions:**

Individuals with an illegal drug use disorder and comorbid mood or anxiety disorders are disproportionately likely to report unmet need for treatment. Integrated mental health and substance use programs could prove effective in addressing their treatment needs.

## Background

According to general population studies, approximately 7-10% of individuals suffer from an illegal drug use related disorder (abuse or dependence) during the course of their lifetime [1]. Up to 53% of individuals with drug abuse or dependence also have other psychiatric disorders [2] and this group disproportionately experiences poor health [3,4], low employment rates [5] and unstable housing [4].

Despite evidence that adequate treatment can reduce the severity and duration of symptoms and harm associated with illegal drug use disorders, only one third of individuals with an illegal drug use disorder receive treatment [5,6], which is less than in the case of other psychiatric disorders such as alcohol abuse or dependence, depression or anxiety disorders [7-12]. Untreated illegal drug use disorders tend to relapse and can result in hospitalizations as well as socio-economic problems [13]. Factors associated with illegal drug use disorder treatment include gender, ethnicity [14], employment status, type of health insurance [15,16], family income, number of drugs used [17] and a criminal record [14]. Access to treatment for illegal drug use disorders is lowest in groups that are older, represent an ethnic minority (particularly African Americans in the United States), are socioeconomically disadvantaged, have restricted medical insurance, and reside in rural areas [14,15,18-20]. In contrast, utilization of treatment for illegal drug use disorders appears to be highest among individuals who use multiple substances [21]. The co-occurrence of illegal drug use disorders with mental health difficulties is associated with an increased likelihood of mental health treatment [22,23]. However, treatment for illegal drug use disorders may be less frequent, particularly in the presence of major depression and anxiety [17,22,23].

Past studies on the role of psychiatric comorbidity with regard to illegal drug use treatment have had a number of limitations. First, they generally examined treatment for alcohol and illegal drug disorders simultaneously, even though treatment rates and associated factors may differ between these two forms of addiction [24]. Second, they focused on the number, rather than types, of psychiatric comorbidities, precluding specific treatment recommendations [21]. Third, they did not study reasons why some individuals with iillegal drug use disorders did not receive treatment despite perceived need [17,21].

In the present study, based upon data from the National Epidemiologic Survey on Alcohol and Related Conditions (NESARC), a nationally representative sample of the US population, we examine the role of comorbid mood and anxiety disorders with regard to treatment for illegal drug use disorders and unmet need for treatment.

## Methods

### Sample and procedures

We used data from the National Epidemiologic Survey on Alcohol and Related Conditions (NESARC). Briefly, this nationally representative study of the US population was conducted in 2001–2002 and 2004–2005 among non-institutionalized adults (≥18 years of age) residing in households and group quarters. African-Americans, Hispanics, and individuals aged 18–24 years were oversampled. All participants were interviewed at home by trained lay interviewers who received extensive training and supervision. After complete description of the study to the participants, written informed consent was obtained. All procedures, including informed consent, received full ethical review and approval from the U.S. Census Bureau and U.S. Office of Management and Budget. Of the 43,093 respondents in 2001–2002, 34,653 were re-interviewed in 2004–2005. The response rate in 2001–2002 was 81% and 86.7% in 2004–2005 [25]. For the present analysis we combined data from 2001–2002 and 2004–2005 and studied individuals with lifetime illegal drug use disorders and their access to treatment. Data were weighted at wave 2 to account for differential loss to follow-up and to be representative of the target population. This analysis includes 34,653 respondents who completed interviews at wave 1 and wave 2.

### Psychiatric disorders

Participants’ lifetime psychiatric disorders were measured using the NIAAA Alcohol Use Disorder and Associated Disabilities Interview Schedule (AUDADIS), a structured interview designed to measure psychiatric disorders and associated conditions in large scale surveys [17] in 2001–2002 and 2004–2005. To meet criteria for a lifetime psychiatric diagnosis participants had to have a positive diagnosis at either NESARC wave (or both). Lifetime illegal drug use disorders were defined as abuse or dependence meeting DSM-IV criteria [26] for any of the following illegal drugs: opioids, sedatives, tranquilizers, amphetamine, cocaine, inhalants/solvents, hallucinogens, cannabis, and heroin.

Lifetime mood disorders were defined as major depression, dysthymia, manic disorder or hypomanic disorder meeting DSM-IV criteria; lifetime anxiety disorders were defined as panic disorder with or without agoraphobia, agoraphobia with no history of panic disorder, social phobia, specific phobia, or generalized anxiety meeting DSM-IV criteria [26].

### Treatment for illegal drug use disorders

We divided the study population into four groups: 1) illegal drug use disorder without comorbid mood or anxiety disorders (n = 244), 2) illegal drug use disorder with comorbid mood disorder only (n = 264), 3) illegal drug use disorder with comorbid anxiety disorder only (n = 110), 4) illegal drug use disorder with comorbid mood and anxiety disorders (n = 496).

### Study outcomes

We examined two study outcomes: treatment for illegal drug use disorders among all those with an illegal drug use disorder and perceived unmet need for treatment among those having not received treatment. Service use was ascertained for each drug of abuse by the following question: “Have you ever gone anywhere or seen anyone for a reason that was related in any way to your use of medicines or drugs - a physician, counselor, Narcotics Anonymous, or any other community agency or professional?” Participants who answered positively were additionally asked to describe the type of treatment they received (outpatient, inpatient, detoxification, rehabilitation, social services). Moreover, participants were asked about perceived unmet need for treatment: “Was there ever a time when you thought you should see a doctor, counselor, or other health professional or seek any other help for your drug use, but you didn’t go?” Participants who met criteria for perceived unmet need for treatment were further asked about reasons for not receiving treatment. Variables ascertaining treatment for illegal drug use disorders and perceived unmet need for treatment were dichotomized. For the purpose of our descriptive analyses participants were split into four groups: 1) those who had an illegal drug use disorder and received treatment (n = 350); those with an illegal drug use disorder who did not receive treatment (n = 764); 2) those who did not receive treatment for an illegal drug use disorder and perceived an unmet need for treatment (n = 127); 3) those who did not receive treatment for an illegal drug use disorder and did not perceive an unmet need for treatment (n = 637).

Additionally, we studied access to treatment for illegal drug use disorders in relation to the type and number of drug of abuse.

### Covariates

Socio-demographic covariates included sex (female vs. male), age (35–54, ≥55 years vs. 18–34 years), educational level (<high school degree vs. ≥ high school), marital status (divorced/separated, widowed, never married vs. married), ethnicity (African American, Other non-Caucasian vs. Caucasian) and family income dichotomized using the lowest quartile of the distribution as the cut-off ($10,000-19000 vs. ≥$20,000/year).

### Statistical analyses

Analyses examining the relationship between the presence of mood and anxiety disorders and a) treatment for an illegal drug use disorder (n = 1114) and b) perceived unmet need for treatment among participants who never received treatment for an illegal drug use disorder (n = 764) were conducted separately.

Because of a significant overlap between treatment for an illegal drug use disorder and treatment for mood (44.5%) and anxiety (28.5%) disorders, in secondary analyses we further controlled for lifetime treatment for mood and anxiety disorders. Furthermore, 74.9% of participants with lifetime illegal drug use disorder also had an alcohol-related disorder; in further analyses we controlled for comorbid alcohol-related disorder.

All analyses were carried out in a logistic regression framework using STATA/SE 12; odds ratios and 95% confidence intervals were weighted to account for sampling design. We used Taylor series estimation methods for variance estimation (STATA ^1^svy^1^ commands) to obtain proper standard error estimates for the cross-tabulations and logistic regressions [27].

## Results

Our study included 1,114 individuals with a lifetime history of an illegal drug use disorder (mean age 37.5 years, SD = 0.45). Table 1 describes characteristics of study participants according to whether they received treatment for an illegal drug use disorder or not, and if not, depending on whether they reported a perceived unmet need for treatment. Overall, participants were mostly male (62.1%), aged 35–54 years (49.2%), Caucasian (71.6%), had graduated from high school (84.0%), had a family income ≥ 20,000 US dollars/year (73.4%), and were not married (52.8%). The majority used one (70.7%), two (17.2%) or three drugs (4.3%). The most frequently used drugs were cannabis (47.2%) and cocaine (22.6%). 21.2% of participants had a comorbid mood disorder only, 11.8% a comorbid anxiety disorder only, and 45.9% comorbid mood and anxiety disorders.

**Table 1:** Characteristics of individuals with a lifetime illegal drug use disorder: the NESARC, 2001/2002-2004/2005 (weighted %)

	**Total study population (n = 1114)**			**Untreated study population (n = 764)**		
	**Treated (n = 350)**	**Not treated (n = 764)**	**P-value**	**Perceived unmet need for treatment (n = 127)**	**No unmet need for treatment (n = 637)**	**P-value**
**Sex**						
Male	64.7	60.9	0.32	61.6	57.5	0.48
Female	35.3	39.1		38.4	42.5	
**Age**						
18-34	36.0	46.5	0.08	49.0	33.1	0.09
35-54	54.3	47.0		44.4	60.7	
≥55	9.7	6.5		6.6	6.2	
**Education**						
<High school	14.4	16.5	0.39	16.7	15.4	0.93
≥ High school	85.6	83.5		83.3	84.6	
**Family income**						
$10 000–19,000	30.3	25.0	0.44	25.0	25.0	0.99
≥$20 000	69.7	75.0		75.0	75.0	
**Marital status**						
Married	51.0	45.6	0.16	44.5	51.4	0.29
Not married	49.0	54.4		55.5	48.6	
**Ethnicity**						
White	75.9	69.7	0.32	69.5	79.9	0.19
Black	10.3	10.8		9.9	15.2	
Other	13.8	19.5		20.6	38.8	
**Type of illegal drug used**						
Cannabis	50.6	48.0	0.73	53.9	42.0	0.06
Cocaine	45.5	30.6	0.0003	27.8	45.2	0.003
Heroin	7.6	2.6	0.008	2.6	2.6	0.99
Tranquilizers	15.8	8.2	0.006	8.1	9.0	0.78
Opioids	20.7	17.6	0.35	17.9	15.8	0.66
Amphetamine	25.2	19.3	0.08	17.7	28.2	0.03
Sedatives	13.2	8.9	0.08	8.8	9.3	0.89
Hallucinogens	12.1	8.9	0.23	8.9	8.9	0.99
Inhalants/Solvents	2.73	1.4	0.28	1.5	0.9	0.59
**Psychiatric profile**						
Illegal drug use disorder + neither mood nor anxiety disorder	18.7	22.0	0.28	24.3	10.2	0.004
Illegal drug use disorder + mood disorders only	20.9	21.3	0.88	21.8	18.7	0.49
Illegal drug use disorder + anxiety disorders only	8.9	13.1	0.10	12.6	15.4	0.53
Illegal drug use disorder + mood and anxiety disorder	51.6	43.6	0.03	41.3	55.7	0.02

### Psychiatric profile classification

As shown in Table 2, the most frequent types of treatment were a consultation with a physician, psychiatrist, psychologist or social worker (46.9%) and attendance of a drug rehabilitation program (10.2%).

**Table 2:** Type of treatment for illegal drug use disorders received, the NESARC, 2001/2002-2004/2005 (n = 350, weighted %)

**Types of treatment**	**N**	**%**
Physician, psychiatrist, psychologist or social worker	258	46.9
Any other agency or professional	81	11.0
Drug rehabilitation	73	10.2
12-Step meeting	45	5.8
Clergy	33	5.1
Did not report the type of treatment	28	5.1
Emergency room	24	3.4
Halfway house	23	3.1
Employee assistance program	20	2.6
Outpatient clinic	14	1.9
Family or social services	11	1.8
Inpatient ward of psychiatric/general hospital or community mental health	9	1.1
Drug detoxification ward or clinic	5	1.0
Methadone maintenance program	4	0.6
Crisis center	3	0.4

Overall, 31.4% of study participants ever received any form of treatment for an illegal drug use disorder. As shown in Table 3, the likelihood of receiving such treatment was not associated with comorbid mood and anxiety disorders. The probability of illegal drug use disorder treatment was highest in participants who were older than 35 years and lowest in participants who were Native American, Asian, or Hispanic.

**Table 3:** Treatment for illegal drug use disorders in relation to comorbid mood or anxiety disorders, the NESARC, 2001/2002-2004/2005, n = 1114 (weighted OR, 95% CI)

	**Bivariate OR**	**95% CI**	**Adjusted OR**^**a**^	**95% CI**
**Psychiatric profile**				
Illegal drug disorder + neither mood nor anxiety disorder	1		1	
Illegal drug disorder + mood disorder only	1.15	0.70-1.90	1.21	0.73-2.01
Illegal drug disorder + anxiety disorder only	0.80	0.43- 1.50	0.75	0.40-1.40
Illegal drug disorder + mood and anxiety disorder	1.39	0.94- 2.05	1.47	0.98- 2.19
**Sex**				
Male	1		1	
Female	0.50	0.43-0.67	0.73	0.51-1.03
**Age**				
18-34	1		1	
35-54	1.01	0.79-1.29	1.46	1.03- 2.06
≥55	0.26	0.18-0.36	1.97	1.09-3.56
**Educational level**				
<High school	1		1	
≥ High school	0.97	0.72-1.29	1.29	0.77-2.16
**Family income**				
$10 000–19,000	1		1	
≥$20 000	1.75	1.41- 2.18	1.52	0.99- 2.35
**Marital status**				
Married	1		1	
Not married	1.65	1.34-2.04	0.79	0.57-1.11
**Ethnicity**				
White	1		1	
Black	0.89	0.70- 1.14	0.93	0.59-1.47
Other	0.82	0.63 -1.07	0.65	0.41-0.02

^a^Odds ratios are weighted to account for sampling design and adjusted for all covariates described in the table.

In additional analyses we observed that the majority of participants who received treatment for illegal drug use disorders used one (33.0%) or two drugs (14.0%) and the number of drugs used was positively associated with treatment (p < 0.01). Moreover, treatment was more frequent among participants with a lifetime history of an alcohol-related disorder (multivariate OR: 1.93, 95% CI 1.34- 2.78), as well as among those who received treatment for mood disorders (multivariate OR: 1.72, 95% CI 1.13-2.61) or anxiety disorders (multivariate OR: 1.89, 95% CI 1.16-3.08).

### Perceived unmet treatment need

Eleven percent of study participants who did not receive treatment reported unmet need for treatment for an illegal drug use disorder. As shown in Table 4, individuals who simultaneously had an illegal drug use disorder and a mood or anxiety disorder had higher levels of perceived unmet need for treatment than participants with no such comorbidities (bivariate ORs respectively: illegal drug use and mood disorder: 2.17, 95% CI: 1.22-3.85; illegal drug use and anxiety disorder: 2.37, 95% CI: 1.25-4.48; illegal drug use and mood and anxiety disorder: 2.74, 95% CI 1.66- 4.55). Other factors significantly associated with perceived unmet need for treatment included being African-American and between 35 and 54 years of age.

**Table 4:** Unmet need for illegal drug use disorders treatment in relation to comorbid mood and anxiety, NESARC, 2001/2002-2004/2005, n = 764 (weighted OR, 95% CI)

	**OR bivariate**	**95% CI**	**Adjusted OR**^**b**^	**95% CI**
**Psychiatric profile**				
Illegal drug disorder + no mood or anxiety disorder	1		1	
Illegal drug disorder + mood disorder only	2.17	1.22- 3.85	2.21	1.23- 4.10
Illegal drug disorder + anxiety disorder only	2.37	1.25- 4.48	2.38	1.27-4.45
Illegal drug disorder + mood and anxiety disorder	2.74	1.66- 4.55	2.90	1.71-4.94
**Sex**				
Male	1		1	
Female	0.54	0.43- 0.67	0.87	0.59-1.29
**Age**				
18-34	1		1	
35-54	1.22	0.94-1.57	1.83	1.3- 2.57
≥55	0.17	0.11-0.27	1.36	0.71- 2.59
**Educational level**				
<High school	1		1	
≥ High school	0.91	0.63-1.31	1.44	0.77- 2.70
**Family income**				
$10 000–19,000	1		1	
≥$20 000	1.53	1.17- 2.001	1.21	0.77-1.9
**Marital status**				
Married	1		1	
Not married	1.61	1.24- 2.08	1.02	0.72-1.45
**Ethnicity**				
White	1		1	
Black	1.17	0.86-1.59	1.63	1.02-2.6
Other	0.98	0.73-1.32	0.96	0.61-1.52

^b^Odds ratios are weighted to account for sampling design and adjusted for all covariates described in the table.

After controlling for all socio-demographic covariates, comorbid mood and anxiety disorders were associated with an elevated likelihood of perceived unmet need for treatment (multivariate ORs respectively: illegal drug use and mood disorder: 2.21, 95% CI, 1.23- 4.10; illegal drug use and anxiety disorder: 2.38, 95% CI, 1.27-4.45; illegal drug use and mood and anxiety disorder: 2.90, 95% CI, 1.71-4.94). After adjusting for treatment for mood and anxiety disorders, individuals who had an illegal drug use disorder as well as an anxiety disorder or both mood and anxiety disorders had higher levels of perceived unmet need for treatment than participants with no such comorbidities (multivariate ORs respectively: illegal drug use and anxiety disorder: 2.34, 95% CI: 1.23- 4.44; illegal drug use and mood and anxiety disorders: 1.94, 95% CI: 1.09- 3.47).

After additionally controlling for lifetime alcohol-related disorder, only comorbid anxiety disorder remained significantly associated with an elevated likelihood of perceived unmet need for treatment (multivariate OR 2.02, 95% CI, 1.03-3.94).

Main reasons for not using treatment services were: the drug problem not being serious enough (37.3%), no willingness to seek treatment (20.0%), spontaneous recovery (11.3%) and social stigma (7.7%) (not shown). These did not differ between the three comorbidity groups (p = 0.15). The majority of participants who reported perceived unmet need for treatment used one (39.8%) or two (16.5%) drugs, primarily cannabis (40.5%), cocaine (27.4%), or heroin (7.4%). The number of drugs used was positively associated with perceived unmet need for treatment (p <0.01).

## Discussion

### Main findings

Our study, based on a nationally representative sample of the US adult population, shows that among individuals who have an illegal drug use disorder, comorbid mood and anxiety disorders are not associated with treatment for illegal drug use disorders. However, common psychiatric comorbidities predict an over twofold increase in perceived unmet need for treatment, which is not accounted for by socio-demographic characteristics, treatment for mood and anxiety disorders and comorbid alcohol use disorder. Overall, these findings suggest that individuals who concomitantly have an illegal drug use disorder and mood or anxiety disorders require special attention from mental health and addiction specialists; integrated mental health and substance use programs could prove effective in addressing their treatment needs.

### Treatment for illegal drug use disorders

Our result of no association between comorbid mood and anxiety disorders and treatment for illegal drug use disorders contrasts with findings of studies which simultaneously examined illegal drug use and alcohol-related disorders [21]. However, in line with prior research, we found that treatment for illegal drug use disorders was associated with the number of drugs used [21], suggesting that the severity of substance use problems is a relevant predictor of treatment seeking.

### Perceived unmet need for treatment

Mood and anxiety disorders were associated with high levels of unmet need for illegal drug use disorder treatment. Overall, the magnitude of the association was similar whether the comorbid disorder was a mood or an anxiety disorder, suggesting that it is the presence of comorbidity rather than its type that is especially relevant. After controlling for treatment for mood and anxiety disorders as well as comorbid alcohol use disorder, only anxiety disorders were associated with an approximately two-fold increase in the likelihood of perceived unmet need for treatment. One possible explanation of this finding is that individuals who simultaneously have illegal drug use problems and mood disorders seek treatment for their mood disorder, because of lesser stigma, easier access, and simpler reimbursement schemes [21]. This does not appear to be the case of participants with comorbid anxiety disorders, who are less likely to seek mental health treatment.

Elevated rates of perceived unmet need for treatment for illegal drug use related disorders may be due to the scarcity of specialized services, absence of adequate programs for simultaneous treatment of co-occurring illegal drug use and psychiatric disorders, as well as stigma. Moreover, perceptions of the severity of the drug use problem and of treatment effectiveness may also play a role. Currently available treatment services for people who have illegal drug use problems other than crack/heroin may not be sufficient, leading to low access and high perceived unmet need for treatment. Furthermore, when illegal drug use disorders are not addressed by mental health services not specialized in addiction, individuals may have the impression that their drug use problems are not serious enough to require treatment or that adequate treatment is not available.

### Limitations and strengths

We need to acknowledge several study limitations. First, illegal drug use and psychiatric disorders, as well as treatment for illegal drug use disorder and perceived unmet need for treatment were studied over participants’ lifetime and we are not able to assess trajectories of substance use and treatment. Research shows that the association between addictive behaviors and mood and anxiety disorders is bidirectional, but common psychiatric disorders most frequently precede onset of substance dependence [28]. Thus, in our study mood and anxiety disorders probably preceded treatment for illegal drug use disorders. Second, data on illegal drug use were collected by federal employees and could be underreported. Nonetheless, the NESARC study interviews were confidential and the reliability of the AUDADIS-IV is good to excellent, therefore the influence of information bias on our measures should be limited [29-31]. Prior to fieldwork, Census employees received extensive training in the AUDADIS, which was created to be administered by non-clinicians, similar to several standardized epidemiological interviews [30]. Moreover, the reliability of the AUDADIS have previously been verified and described [31]. Third, access to treatment for illegal drug use disorders and unmet need for treatment were ascertained using a single measure specific to the NESARC study, previously used by other researchers [32]. Fourth, the study population did not include individuals who are institutionalized, who may have elevated rates of both illegal drug use disorders and unmet treatment needs. Therefore, the association between mood and anxiety disorders and unmet need for illegal drug use treatment may be stronger than we report. Fifth, we were not able to examine whether access to treatment for illegal drug use disorders varied between rural and urban areas, by state, or type of treatment unit.

Our study also has strengths. First, we studied a nationally representative sample of the US adult population which limits the possibility of selection bias. Second, psychiatric disorders were assessed using a structured diagnostic questionnaire, which evaluates the presence of clinically relevant disorders as measured by DSM-IV criteria [26]. Third, we investigated factors associated with service use, but also unmet perceived need for treatment for illegal drug related disorders.

### Integrated treatment of comorbid illegal drug disorders and mental health problems

National surveys indicate that only half of US mental health centers offer treatment programs for individuals with ‘dual diagnosis’ and this proportion did not significantly change during the past decade [6]. Lack of progress in this area can be explained by a) insufficient clinical knowledge, b) the absence of standardized screening procedures to identify individuals who have an illegal drug use disorder [33,34], and c) incomplete treatment guidelines in case of dual diagnosis and insufficient organizational support [35]. Patients themselves repeatedly report that illegal drug use disorders are often ignored and not treated by mental health care providers.

According to a proposed four-quadrant continuity-of-care model, a) persons with low severity psychiatric and substance use disorders should be treated in the primary health care system (quadrant I); b) persons with high severity psychiatric disorders and low severity substance use disorders should be treated in the mental health system (quadrant I); c) persons with low severity psychiatric disorders and high severity substance use disorders should be treated in the addiction treatment system (quadrant III); d) persons with high severity psychiatric and substance use disorders need to use multiple treatment systems and make more frequent use of emergency and inpatient services (quadrant IV) [36]. This relies on a well-functioning referral and collaboration system between the point-of-contact (primary care, emergency departments, etc.) and specialized care. Number of studies conducted in the last 15 years provided evidence for the effectiveness of integrated treatment for comorbid psychiatric and substance use disorders [37-39]. Nevertheless the integration of mental health and substance use treatment services has produced uneven results due to a number of barriers such as the absence of regulations, the lack of appropriate financial resources [40], the lack of sufficiently experienced staff [41], high rates of stuff turn over [42], and the lack of tools and implementation strategies [43]. In a context of low availability of integrated treatment, progress has been made with the development and continued validation of external rating tools (DDCAT -the Dual Diagnosis Capability in Addiction Treatment and DDCMHT-the Dual Diagnosis Capability in Mental Health Treatment) to determine the capability of substance abuse and mental health service providers to treat co-occurring disorders [44,45]. Previous evaluations using the DDCAT showed that the majority of substance abuse programs were rated less than ‘capable’ and only few programs achieved or exceeded the ‘capable’ level [46,47]. Overall, growing evidence stresses the importance of routine screening for illegal drug use disorders in patients with other psychiatric disorders and the integration of addiction services into psychiatric and medical treatment settings to promote symptom reduction and stable remission.

## Conclusions

Our study suggests that the presence of comorbid mood and anxiety disorders is associated with an increased likelihood of perceived unmet need for treatment for illegal drug use disorders, which should be brought to clinicians’ attention. The presence of mood and anxiety disorders should increase awareness of a potential illegal drug use disorder which also requires treatment. In terms of public health, our findings imply that integrated treatment services that provide combined treatment for illegal drug use disorders and mental health problems could also help improve treatment of patients with dual diagnosis.

## Competing interests

The authors declare that they have no competing interests.

## Authors’ contributions

MM, PS, NY and SM designed the study. EP undertook scientific literature searches, statistical analyses, and wrote the first draft of the manuscript. MM, NY, PS and SM contributed to data analysis and interpretation and participated in writing subsequent versions of the manuscript. MM was responsible for the finalization of the manuscript. All authors participated in revising the article critically for important intellectual content, read, and approved the final manuscript.

## Pre-publication history

The pre-publication history for this paper can be accessed here:

http://www.biomedcentral.com/1471-244X/14/89/prepub
